# Molecular characterization of the 5′-UTR of retinal dystrophin reveals a cryptic intron that regulates translational activity

**Published:** 2010-12-07

**Authors:** Ikuko Kubokawa, Yasuhiro Takeshima, Mitsunori Ota, Masahiro Enomoto, Yo Okizuka, Takeshi Mori, Noriyuki Nishimura, Hiroyuki Awano, Mariko Yagi, Masafumi Matsuo

**Affiliations:** Department of Pediatrics, Graduate School of Medicine, Kobe University, Kobe, Japan

## Abstract

**Purpose:**

Mutations in the dystrophin (*DMD*) gene cause Duchenne or Becker muscular dystrophy (DMD/BMD). *DMD* contains a retina-specific promoter in intron 29. The short R-dystrophin transcript from this promoter has a retina-specific exon 1 (R1) joined to exon 30 of the *DMD* gene. It has been claimed that this is responsible for the ophthalmological problems observed in DMD/BMD. This research characterizes the structure of the 5′-untranslated region (5′-UTR) of human R-dystrophin.

**Methods:**

The 5′-UTR of the human R-dystrophin transcript was amplified from human retina and 20 other human tissue RNAs by reverse transcription polymerase chain reaction (RT–PCR). Amplified products were identified by sequencing. The translational activities of transcripts bearing differing 5′-UTRs were measured using a dual luciferase assay system.

**Results:**

RT–PCR amplification of the R-dystrophin transcript from the retina using a conventional primer set revealed one product comprising exon R1 and exons 30 to 32 (R-dys α). In contrast, three amplified products were obtained when a forward primer at the far 5′-end of exon R1 was employed for RT–PCR. R-dys α, and a shorter form in which 98 bp was deleted from exon R1 (R-dys β), were the two major products. A minor, short form was also identified, in which 143 bp was deleted from exon R1 (R-dys γ). The two primary retinal products (R-dys α and β) encoded an identical open reading frame. The 98 bp deleted in R-dys β was identified as a cryptic intron that was evolutionarily acquired in higher mammals. The shorter R-dys β was expressed in several tissues with a wide range in expression level, while R-dys α was retina specific. The 5′-UTRs of R-dys α and β were examined for translational activity using a dual luciferase assay system. Unexpectedly, the 5′-UTR of R-dys β showed lower translational activity than that of R-dys α. This lower activity was presumed to be due to the removal of internal ribosome entry sites by activation of cryptic intron splicing.

**Conclusions:**

An evolutionarily-acquired cryptic intron was identified in the 5′-UTR of the human R-dystrophin transcript. The two abundant R-dystrophin transcripts in the retina showed different translational activities in vitro owing to their differential splicing of the cryptic intron. This evolutionarily-acquired alternative splicing may act as a molecular switch that regulates translation of the R-dystrophin transcript.

## Introduction

The dystrophin (*DMD*) gene is responsible for the most common inherited muscle diseases, Duchenne and Becker muscular dystrophies (DMD/BMD). *DMD* is the largest known human gene, spanning more than 2.5 Mb on chromosome Xp21.2, and comprises 79 exons and large introns. At least eight alternative promoters/first exons are scattered among the introns. Four tissue-specific promoters at the 5′-end of the gene express full-length dystrophin, while four internal promoters express smaller isoforms containing unique first exons that are activated in a tissue-specific manner [1–3].

An alternative promoter/first exon located within intron 29 is strongly expressed in the retina. The first exon (R1) splices to exon 30 of the *DMD* gene to encode R-dystrophin [3,4]. Exon R1 is 236 bp long and encodes 13 retina-specific amino acids, leaving 197 bp as the 5′-untranslated region (5′-UTR) [4]. This protein has been identified as human R-dystrophin by western blotting of retinal dystrophin proteins (Dp260) [5]. In addition, a single sequence with a 143-bp deletion in exon R1 has been deposited in GenBank (NM_004011.3). This variant contains a 95 bp exon R1 encoding a unique 16 amino acids of N-terminal end. R-dystrophin has been shown to be involved in the commitment of synaptic maturation and attachment of the retina to the vitreous [6–8]. Furthermore, R-dystrophin has been categorized as a cytolinker in skeletal muscle because it organizes costameric microtubules in the skeletal muscle of transgenic mdx mice expressing R-dystrophin [9].

DMD and BMD have been described to have ophthalmological complications [8,10,11]. A reduced electroretinogram (ERG) b-wave was identified in DMD and considered a direct consequence of dystrophin deficiency [4,5,8,10]. A subset of DMD patients with deletions downstream of exon 30, affecting the splicing and transcription of R-dystrophin, exhibit a red-green color vision defect, while DMD patients who have dystrophin mutations upstream of exon 30 have seemingly normal color vision [11]. This color vision defect is likely caused by a loss of R-dystrophin. Thus, both molecular and clinical findings suggest an important role for R-dystrophin.

Alternative splicing is a source of genetic variation, and more than 95% of genes have at least one alternative splicing site [12]. Alternative splicing within the protein-coding region attracts much attention, because it is likely to directly impact protein function. In contrast, alternative splicing in 5′-UTRs has not been characterized as well. It is known that the 5′-UTR is a major site of translational regulation through internal ribosome entry sites (IRES) [13–15] or upstream AUG (uAUG) motifs [16]. Abnormalities in the 5′-UTR can be pathogenic [13,17]. Alternative splicing in the 5′-UTR has been shown to play a significant role in gene function [18]. However, the 5′-UTR of the human R-dystrophin transcript has not been well characterized.

Here, we identified an evolutionarily-acquired alternative splicing pathway in the 5′-UTR of the R-dystrophin transcript. We showed that transcripts with an alternatively-spliced 5′-UTR have a lower translational activity than those with a non-spliced 5′-UTR.

## Methods

### Transcript analysis

RT–PCR amplification was conducted to examine the R-dystrophin transcript. Human total RNAs from retina and 20 other tissues (adrenal gland, colon, cerebellum, whole brain, fetal brain, fetal liver, heart, kidney, liver, lung, placenta, prostate, salivary gland, skeletal muscle, spleen, testis, thymus, thyroid gland, trachea, and uterus) were obtained from a human total RNA Master Panel II (Clontech Laboratories, Inc., Mountain View, CA). cDNA was synthesized as described previously [19] from 2.5 µg of each total RNA. A retinal promoter-derived transcript spanning exon R1 to exon 32 was PCR amplified using the following primers: forward primer, RdysF-N119: 5′-ATG CAG AGA TCC CTG ATC CTA TAG-3′ [4] and reverse primer on exon 32, 2F: 5′-TTC CAC ACT CTT TGT TTC CAA TG-3′. To extend the amplified region, another forward primer was used at the 5′-end of exon R1 (Rdys-F: 5′-GGA GGA ACA TTC GAC CTG AG-3′). PCR amplification was performed in a total volume of 20 µl, containing 2 µl of cDNA, 2 µl of 10× ExTaq buffer (Takara Bio, Inc., Shiga, Japan), 0.5 U of ExTaq polymerase (Takara Bio, Inc.), 500 nM of each primer and 250 µM dNTPs (Takara Bio, Inc.). Twenty-eight cycles of amplification were performed on a Mastercycler Gradient PCR machine (Eppendorf, Hamburg, Germany) using the following conditions: initial denaturation at 94 °C for 5 min, subsequent denaturation at 94 °C for 0.5 min, annealing at 59 °C for 0.5 min and extension at 72 °C for 1 min. Amplified PCR products were electrophoresed on a 2% agarose gel with a low molecular weight DNA standard (ϕX174-HaeIII digest; Takara Bio, Inc.), and stained with ethidium bromide.

PCR-amplified bands were excised from the gel with a sharp razor, pooled and purified using a QIAGEN gel extraction kit (QIAGEN, Inc., Hilden, Germany), according to the manufacturer’s instructions. Purified products were subcloned into the pT7 blue T vector (Novagen, Inc., San Diego, CA), and sequenced using a Taq dye terminator cycle sequence kit (Life Technologies Corp., Carlsbad, CA) with an automatic DNA sequencer (3130 Genetic Analyzer; Life Technologies Corp.), as described previously [20]. RT–PCR products were semi-quantified using a DNA 1000 LabChip kit on an Agilent 2100 Bioanalyzer (Agilent Technologies, Santa Clara, CA).

To check the integrity and concentration of the cDNA, the glyceraldehyde 3-phosphate dehydrogenase (*GAPDH*) gene was also RT–PCR amplified, as described previously [21].

### Translational activity assay

#### Construction of plasmids

To measure the translational activity of the 5′-UTR of dystrophin transcripts, dual-luciferase reporter assays using the psiCHECK-2 vector (Promega Corp., Madison, WI), which allows simultaneous expression of *Renilla* and firefly luciferase from a single plasmid, were conducted in HEK293 cells. The 5′-UTR sequences of R-dys α (α5′-UTR) and β (β5′-UTR) were inserted into the *Renilla* luciferase gene. Before inserting the 5′-UTR sequences into the psiCHECK-2 vector, the ATG of the *Renilla* luciferase was mutated to TTG, named psiCHECK-2-TTG, so that the *Renilla* luciferase expression would be driven by the primary ATG initiation codon of the gene under investigation. The 5′-UTR sequences, up to and including the primary ATG initiation codon, were PCR amplified. The amplified products were cloned and ligated into the NheI site directly preceding the *Renilla* luciferase gene in the plasmid psiCHECK-2-TTG.

The α5′-UTR was PCR amplified using the primers H-Dp260Ex1-F: 5′-cgc *gct agc* TAA TGA GAT CAG GAG GAA CA-3′ and H-Dp260Ex1-α: 5′-cgc *gct agc* CTC ATT CAG CTC TGT TGA TA-3′; the β5′-UTR was amplified using H-Dp260Ex1-F and H-Dp260Ex1-β: 5′-cgc *gct agc* CTC ATT CAG CTA TTA AGG AA-3′. Upper case letters corresponded to sequences of the 5′-UTR. Lower case letters were sequences attached to create an NheI cut site (*gct agc*).

#### Luciferase activity assay

HEK293 cells were grown at 37 °C in a humidified atmosphere containing 5% CO_2_ in Dulbecco’s modified Eagle’s medium (Sigma-Aldrich Corp., St Louis, MO) supplemented with 10% fetal bovine serum (Life Technologies Corp.) and 1% PSN antibiotic mixture (Life Technologies Corp.). HEK293 cells were seeded at 4×10^5^ cells per well in 12-well dishes. After overnight incubation, the cells grew to approximately 80% confluency and 800 ng of each of the three vector constructs (the unmodified plasmid psiCHECK-2-TTG and the plasmids psiCHECK-2-α and -β) were transfected into the cells using Lipofectamine™ 2000 (Life Technologies Corp.). Twenty-four h after transfection, cells were washed with phosphate-buffered saline and resuspended in Passive Lysis Buffer (Promega Corp.). The firefly and *Renilla* luciferase activities were measured using a dual-luciferase reporter assay kit (Promega Corp.) with a plate reader (Fluoroskan Ascent FL; Thermo Fisher Scientific., Waltham, MA), according to the manufacturer’s instructions. All experiments were performed in triplicate. To account for nonspecific effects on reporter plasmids, experimental results were expressed as a normalized ratio. The ratio of *Renilla* and firefly luciferase activity was normalized against the unmodified plasmid psiCHECK-2-TTG. The relative ratio of *Renilla*/firefly luciferase activity determined from cells transfected with the plasmid psiCHECK-2-α was set at 1 and was compared with that determined from the plasmid psiCHECK-2-β.

### Analysis

The probability scores for the splice acceptor and donor sites were calculated as described previously [22]. Statistical significance was examined using Student’s *t* test using GraphPad Prism software, version 5.02 (GraphPad Software Inc., San Diego, CA). A significant difference was described, if the p value was less than 0.05. The secondary structure of the 5′-UTR was analyzed using Mfold, version 3.2. Internal ribosome entry sites (IRESs) were analyzed using IRESite.

## Results

### Retinal dystrophin transcript

**Figure 1:**
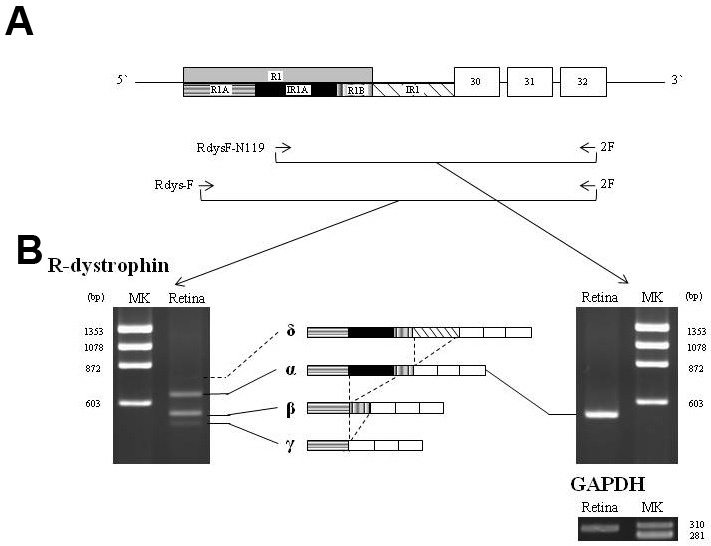
Reverse transcription polymerase chain reaction (RT–PCR) amplification of the R-dystrophin transcript. **A**: Schematic description of the region encompassing exons R1 to 32 of the dystrophin (*DMD)* gene. RT–PCR primers (RdysF-N119, Rdys-F and 2F) are shown as horizontal arrows. Boxes and bars indicate exons and introns, respectively. Exon/intron structures are described according to previous studies and the present study above and below the line, respectively. In this study, exon R1 is divided into three segments: the boxes labeled R1A (horizontal-lined), IR1A (black) and R1B (vertical bold-lined) represent exon R1A (93 bp), intron R1A (98 bp) and exon R1B (45 bp), respectively. Intron R1 was also incorporated into the mRNA (oblique-lined box). Numbers within the boxes indicate the exon number. The actual lengths of exon R1 and intron R1 are 236 bp and 105 bp, respectively. **B**: RT–PCR-amplified products of the fragment encompassing exons R1 to 32. Amplification from the retina using the conventional primer RdysF-N119 revealed one clear band of 529 bp (R-dys α; right). In contrast, RT–PCR amplification using the outer forward primer Rdys-F revealed three bands: two major products of 636 bp and 538 bp (R-dys α and β, respectively) and one weak product of 493 bp (R-dys γ; left). In addition, sequencing of subclones revealed an additional, larger product (R-dys δ). The exon structure of each product is described in the middle of each panel. The four R-dystrophin transcripts are shown by four bars. A fragment of *GAPDH* (302 bp) was amplified as a control (bottom). MK refers to the size marker (φX174 HaeIII digest).

To characterize the 5′-UTR of the R-dystrophin transcript, a region from exon R1 to exon 32 was RT–PCR amplified from human retina RNA using a conventional forward primer on exon R1 (RdysF-N119) and a reverse primer on exon 32 (2F). One clear PCR-amplified product was obtained that comprised exons R1, 30, 31, and 32 (R-dys α) (Figure 1), as described previously [4]. This agreed with the current understanding of retinal dystrophin [11].

To examine the full length of exon R1, the same region was amplified using an outer forward primer (Rdys-F), 107 bp upstream of RdysF-N119 and located at the far 5′-end of exon R1. Three amplified products were visualized on agarose gels, with two major products and one minor product (Figure 1). Among the two major products, the longer product was identical to R-dys α. The shorter major product had the same exon content as R-dys α but had a 98-bp deletion within exon R1 located 45 bp upstream of the 3′-end of exon R1 (R-dys β; Figure 1). R-dys β was a novel variant of the R-dystrophin transcript. Because the ATG start codon is present within the common 45-bp region, the translational reading frame of both R-dys α and β was identical. R-dys β was expressed at a similar level to R-dys α in the retina, and it was speculated that the R-dys β may be physiologically important. The minor PCR band was identified to have a 143-bp deletion in exon R1 that shortened it to 93 bp (R-dys γ; GenBank NM_004011.3). In addition to the three visible products, a clone of 741 bp was identified by sequencing of subclones. This clone retained a 105-bp intron R1 between exons R1 and 30 (R-dys δ; Figure 1). This is suggested to be an immature mRNA transcribed from the R-dystrophin promoter.

### Alternative splicing of the 5′-UTR of R-dystrophin

**Figure 2:**
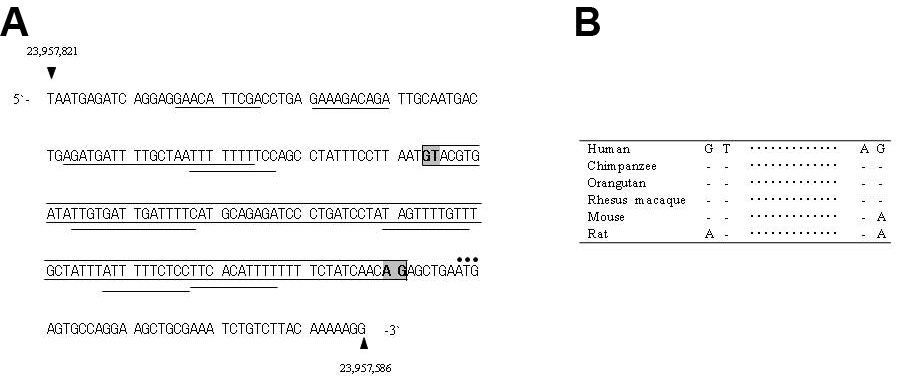
Genomic sequence of exon R1. **A**: Features of exon R1 (236 bp; GenBank NW_001842360.1). The 98-bp sequence, deleted in some transcripts and presumed to be a cryptic intron, is marked by a box. This sequence starts with GT and ends with AG (shaded). Bars below the sequence indicate internal ribosome entry sites. Dots over the ATG codons indicate a translation start site. **B**: Consensus dinucleotide sequences for the cryptic intron. The AG and GT splice consensus dinucleotides are present in humans and other mammals, including chimpanzee, orangutan and rhesus macaque. The mouse and rat genomic sequences contain GT/AA and AT/AA, respectively.

The difference between the two major retinal transcripts (R-dys α and β) was the absence or presence of the 98-bp sequence from their 5′-UTRs. Examination of this sequence revealed GT and AG dinucleotides at its 5′- and 3′-end, respectively (Figure 2). The probability scores for these dinucleotides to act as splice donor (GT) or acceptor (AG) sites were calculated as 0.76 and 0.93, respectively. These values were within the ranges of scores for the rest of *DMD* [23]. It was concluded that the 98-bp region is a novel cryptic intron embedded within exon R1. As a result, the 236-bp exon R1 can be divided into three segments: the 93-bp exon R1A, the 98-bp intron R1A and the 45-bp exon R1B (Figure 1 and Figure 2). The difference between R-dys α and β was thus caused by alternative splicing of intron R1A.

Alternative splicing of intron R1A has not been described in previous reports [4, 24]. It was found that the cryptic intron R1A was evolutionarily acquired in humans but is not present in rodents, in which intensive studies on R-dystrophin have been conducted [25]. The mouse and rat genomic sequences corresponding to the human donor and acceptor sites of intron R1A have GT/AA and AT/AA, respectively, compared with GT/AG in humans (underlining indicates a mismatched nucleotide; Figure 2). Evolutionarily higher animals, including the chimpanzee, orangutan and rhesus macaque, have the same sequence as humans in this region (Figure 2).

### Tissue distribution of R-dystrophin variants

**Figure 3:**
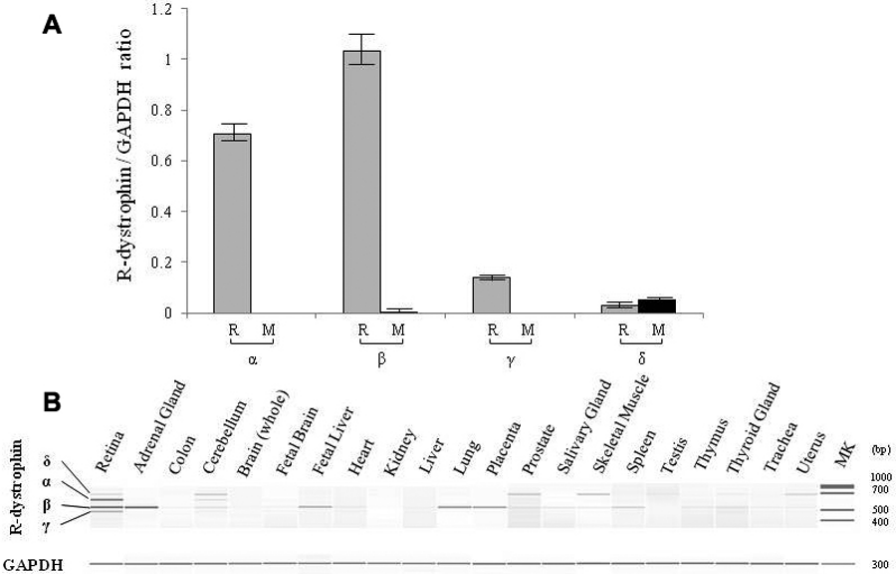
R-dystrophin variants in human tissues. **A**: Semi-quantitative analysis of R-dystrophin variants in the retina and the skeletal muscle. The ratio of each variant/GAPDH in the retina (R) and the skeletal muscle (M) are shown. A fragment comprising exons R1 to 32 was RT–PCR amplified from the retina and the skeletal muscle and semi-quantitated. The value was obtained by three quantifications and shown as mean±SD. In the retina (R), strong expression of R-dys α (α) and β (β) was observed. However, no significant difference between R-dys α and β was observed (0.70±0.05 and 1.03±0.16, respectively). In the skeletal muscle (M), R-dys α, β and γ (γ) were not detected but R-dys δ (δ) was observed at the quite low level. **B**: RT–PCR amplified products are shown. A fragment comprising exons R1 to 32 was RT–PCR amplified from 21 human tissues. Four products were obtained from the retina (R-dys α, β, γ, and δ) but R-dys α and β were the main products. R-dys α was also identified weakly in the cerebellum. R-dys β was present in several tissues including the adrenal gland. MK refers to the size marker (DNA 1000 Markers).

To examine the tissue expression patterns of the four variants of the R-dystrophin transcript, RT–PCR amplification using an outer forward primer was conducted in 21 human tissue RNAs. Semi-quantitative analysis was conducted in the retina and the skeletal muscle where retina- and muscle-specific promoters were active, respectively (Figure 3). In the retina, four variants could be detected, but R-dys α and β were the two main products. Expression levels of R-dys α and β were not significantly different. In the skeletal muscle, R-dys α, β, and γ were not detected but R-dys δ was marginally amplified. Unexpectedly, R-dys β, the shorter retinal product, was clearly amplified not only in the retina but also in the adrenal gland (Figure 3). Furthermore, R-dys β was detected weakly in the fetal liver, the lung, the placenta and the spleen. Expression of R-dys α was observed strongly in the retina and weakly in the cerebellum but not in any other tissues. It is interesting that R-dys α was detected weakly in the cerebellum (Figure 3) because R-dys α has been proposed to have a neuron-specific function [6–8].

### Translational differences mediated by the 5′-UTRs of R-dys α and β

**Figure 4:**
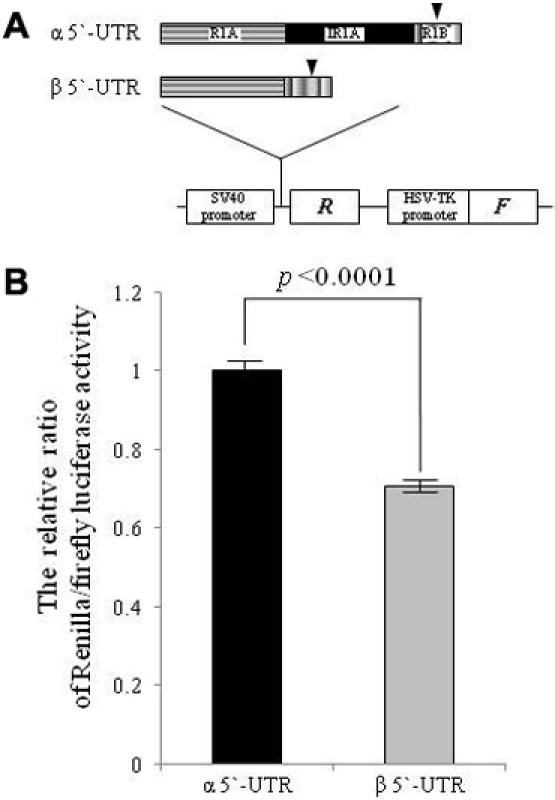
Translational activity of the 5′-UTR. **A**: Structure of the expression vector and inserted sequence. The psiCHECK-2 PCR vector is described (bottom). This vector contains the *Renilla* (*R*) and firefly (*F*) luciferases as the upstream and downstream cistrons, respectively. Both the α5′-UTR and the β5′-UTR were amplified (upper and middle, respectively) and inserted into the cloning site. Before inserting the 5′-UTR sequences into the psiCHECK-2 vector, the ATG of the *Renilla* luciferase was mutated to TTG, so that the *Renilla* luciferase expression would be driven by the primary ATG initiation codon (reverse triangle) of the gene under investigation. **B**: Luciferase activity. Three vector constructs (the unmodified plasmid psiCHECK-2-TTG and plasmids psiCHECK-2-α and -β) were transfected into HEK293 cells, and the firefly and *Renilla* luciferase activities were measured 24 h after transfection. All experiments were performed in triplicate. The ratio of *Renilla* and firefly luciferase activity was normalized against the unmodified plasmid psiCHECK-2-TTG. The relative ratio of *Renilla*/firefly luciferase activity determined from cells transfected with the plasmid psiCHECK-2-α was set at 1. The relative ratio of *Renilla*/firefly luciferase activity was significantly lower for the β5′-UTR than for the α5′-UTR (0.71±0.01 and 1.00±0.01, respectively).

Although R-dys α and R-dys β encode an identical protein, they may be differentially translated in human tissues, because of their differing 5′-UTRs (α5′-UTR and β5′-UTR, respectively). Using a dual luciferase assay system, the translational activity of the β5′-UTR was significantly lower than that of the α5′-UTR (0.71±0.01 versus 1.00±0.01) (Figure 4). It was concluded that R-dys β is translated at a lower level than R-dys α, even though it is more widely expressed. Thus, R-dys α was considered more important in regards to protein production.

To investigate the mechanism providing the translational difference between R-dys α and β, their 5′-UTRs were examined for upstream open reading frames (uORFs) [26,27] or secondary structure [28], but no clear explanation was obtained. When IRESs, which are considered to regulate translation [29,30], were analyzed, four IRES motifs were identified within the 98-bp intron R1A, and four were identified within the 93-bp exon R1A (Figure 2). Because intron R1A was rich in IRES motifs, R-dys β, which lacks intron R1A, was considered weak for translational activity.

## Discussion

In this study of the 5′-UTR of the R-dystrophin transcript, four different transcripts were identified in human retina (Figure 1). Two (R-dys α and γ) were previously known (GenBank, NM_004012.3 and NM_004011.3, respectively), and two new transcripts (R-dys β and δ) were cloned. Alternative splicing was shown to provide diversity in the transcripts produced from the retinal promoter, and a new, evolutionarily-acquired intron was identified within exon R1 (Figure 2). Among the four transcripts, two (R-dys α and β) were the primary products in the retina (Figure 1); these encode an identical open reading frame but have different 5′-UTRs, depending on the pattern of intron R1A splicing. It is proposed that splicing of the cryptic intron R1A in the 5′-UTR is a key regulator of the physiologic roles of these variants.

Considering that R-dys β was expressed in several human tissues (Figure 3), the production of R-dys β was considered the default pathway for the R-dystrophin transcript. It is supposed that an unidentified retina-specific factor shifts this default pathway to include the cryptic intron R1A in the retina, thereby producing R-dys α. R-dys α has a higher translational activity than R-dys β, at least in vitro (Figure 4), which produces R-dystrophin in the retina.

Intron R1A was found to have been acquired during evolution between rodents and primates (Figure 2), suggesting a higher physiologic role for the alternative splicing of cryptic intron R1A. Important roles for alternative splicing in the 5′-UTR have been demonstrated in the regulation of translation in certain human genes [12,13,18,27]. The β5′-UTR, without intron R1A, showed lower translational activity than the α5′-UTR in a dual luciferase assay (Figure 4). This result is contrary to the understanding that a long 5′-UTR suppresses translation by increasing the energy that a navigating ribosome needs to reach the AUG through a highly structured 5′-UTR, stable secondary structures or multiple uORFs [17]. Intron R1A retained in the α5′-UTR seemed to increase translation of R-dys α by virtue of multiple IRESs (Figure 4). It is known that mRNAs encoding proteins involved in cell growth, proliferation and apoptosis have structured 5′-UTRs that harbor IRESs [13]. Therefore, R-dys α retaining many IRESs is supposed to have an important physiologic role. Although an ERG abnormality and a red-green color vision defect have been identified in DMD [11,31], the two variants identified in the retina are considered to explain these abnormalities. Further studies analyzing mutations in the 5′-UTR region are required to clarify this.

R-dys α was expressed in the retina and weakly in the cerebellum (Figure 3). This may correlate with synaptic junction formation in these tissues [7,32–35]. No clear abnormalities in the cerebellum of DMD patients have been reported; however, it is possible that a cerebellar phenotype may be revealed as a more precise examination of cerebellar function becomes possible, or when DMD patients can survive for longer than at present. One third of DMD patients show mental retardation [36], but direct genotype-phenotype correlations have not been established for mental retardation in DMD [37]. It would be interesting to analyze alternative splicing of R-dystrophin in relation to mental retardation in DMD.

R-dys β was produced by activated splicing of both introns R1A and R1 from the transcript. This suggests that the same splicing factors facilitate the splicing of both introns. Conversely, in the retina, an unidentified splicing factor may inhibit splicing of intron R1A. Clarification of these factors may facilitate understanding of the physiologic roles of the two variants. The pattern of intron R1A alternative splicing was shown to be key in determining translational activity (Figure 4). IRESs have been reported to provide molecular switches, allowing maintenance of the expression of proteins essential for cell survival or death [13]. Thus, the alternative splicing of intron R1A may act as a molecular switch regulating the expression of R-dystrophin in the retina. The evolution of alternative splicing in the 5′-UTR has been proposed to contribute to the regulation of translation [18]. Our results add another example of an evolutionarily-acquired alternative splicing pathway that regulates translational activity.
